# Regulation of trophic factors in the choroid plexus of aged mice

**DOI:** 10.21203/rs.3.rs-4123786/v1

**Published:** 2024-03-22

**Authors:** Jayanarayanan Sadanandan, Monica Sathyanesan, Samuel S Newton

**Affiliations:** University of South Dakota; University of South Dakota; University of South Dakota

**Keywords:** Choroid plexus, Blood-cerebrospinal fluid barrier, Tight junctional proteins, Neurotrophic factors, Aging, epithelial cells, Klotho, Cerebrospinal fluid, Vascular Endothelial Growth Factor

## Abstract

**Background:**

The choroid plexus (CP) is an understudied tissue in the central nervous system (CNS), primarily implicated in cerebrospinal fluid (CSF) production. Additionally, CP produces numerous neurotrophic factors (NTF), which circulate to different regions of the brain. Regulation of NTF in the CP during natural aging has yet to be discovered. Here, we investigated the age and gender-specific transcription of NTFs along with the changes in the tight junctional proteins (TJPs) and water channel protein Aquaporin (AQP1).

**Methods:**

We used male and female mice for our study. We analyzed neurotrophic factor gene expression patterns using quantitative and digital droplet PCR at three different time points: mature adult, middle-aged, and aged. Additionally, we used immunohistochemical analysis (IHC) to evaluate in vivo protein expression. We further investigated the cellular phenotype of these NTFS, TJP and water channel proteins in the mouse CP by co-labeling them with the classical vascular marker, Isolectin B4, and epithelial cell marker, plectin.

**Results:**

Aging significantly altered the NTF’s gene expression in the CP Brain-derived neurotrophic factor (BDNF), Midkine, VGF, Insulin-like growth factor (IGF1), IGF2, klotho, Erythropoietin, and its receptor were reduced in the aged CP of males and females. Vascular endothelial growth factor (VEGF) transcription was gender-specific; in males, gene expression is unchanged in the aged CP while females showed an age-dependent reduction. Age-dependent changes in VEGF localization were evident, from vasculature to epithelial cells. IGF2 and klotho localized in the basolateral membrane of the CP and showed an age-dependent reduction in epithelial cells. Water channel protein AQP1 localized in the tip of epithelial cells and showed an age-related reduction in mRNA and protein levels. TJP’s JAM, CLAUDIN1, CLAUDIN2, and CLAUDIN5 were reduced in aged mice.

**Conclusions:**

Our study highlights transcriptional level changes in the CP during aging. The age-related transcriptional changes exhibit similarities as well as gene-specific differences in the CP of males and females. Altered transcription of the water channel protein AQP1 and TJPs could be involved in reduced CSF production during aging. Importantly, reduction in the neurotrophic factors and longevity factor Klotho can play a role in regulating brain aging.

## Background

The blood-cerebrospinal fluid barrier (BCSFB) is a physicochemical barrier established by choroid plexus (CP) epithelial cells in the lateral, third, and fourth cerebral ventricles. CP is composed of highly specialized cuboidal epithelium that is continuous, with ependymal cells lining the ventricles of the brain. These cuboidal epithelial cells are interconnected by tight junctions on their apical surface along with a core of fenestrated capillaries, allowing the filtration of plasma [1]. Besides their barrier function, choroid plexus epithelial cells have a secretory function by producing 70–80% of cerebrospinal fluid (CSF) [2]. With the support of fenestrated capillaries and elevated blood flow, the cuboidal epithelial cells provide the brain with a high turnover rate of fluid containing hormones, peptides, and micronutrients to the neuronal network [3, 4, 5].

The structure of the CP lends itself to involvement in an extensive spectrum of physiological actions on the brain. In addition to secretory functions, the CP also performs excretory functions, such as the removal of toxic peptides from the brain through various transporters [6]. Maintaining epithelial-tight junctional integrity, maintained by tight junctional proteins, is necessary to protect the central nervous system (CNS). Junctional proteins CLDN1, CLDN2, CLDN11, occludin, the zonula occludens protein (ZO-1), and Junctional adhesion molecules (JAM) are present in tight junctions of the choroid plexus epithelium [7, 8, 9, 10]. Claudins and occludin are the major transmembrane molecules facilitating epithelial contact [11]. The importance of apical tight junctions in the CP epithelium has been neglected, and studies still need to be done regarding the expression of the choroid epithelial tight junction proteins in the later stages of life.

The choroid plexus plays an essential role in supporting neuronal function by producing a large variety of trophic factors during embryonic and adult stages [12, 13]. In the adult brain, CP secretes major neurotrophic factors, including fibroblast growth factors, epithelial growth factors, platelet-derived growth factors, insulin-like growth factors, vascular endothelial growth factors, midkine, and BDNF to the CSF, which circulates to the different parts of the brain, maintaining neuronal and vascular function [14, 15, 16, 17, 18, 19]. Most of these trophic factors are associated with either adult neurogenesis, plasticity, cognition, or angiogenesis [17, 20, 21, 22]. Growth factors like VEGF and TGF-b are known to be involved in the maintenance of the choroid plexus [1, 23].

Natural aging affects the structure and function of the choroid epithelial cells. Aged choroid epithelial cells exhibit increased pathological protein deposits called Biondi ring tangles [24, 25] with depleted glucose metabolism and energy production [26, 27]. During natural aging, cranial and ventricular CSF volume doubles [28, 29, 30], and this increase in CSF volume, coupled with the reduction in CSF production and secretion, slows the turnover rate of CSF by three to four-fold [31, 32]. This disruption in CSF turnover can contribute to the etiology of age-related neurocognitive disorders [33, 34, 35, 36, 37].

Studies have shown that aging alters levels of several cerebrospinal fluid (CSF) protein levels [38]. Dysregulated protein expression could be due to age-related dysfunction of the BCSF barrier. In an aged brain, the permeability of the BCSF barrier integrity is compromised, and the proteins contained in the CSF may be produced from the brain and CP Although the CP is known to produce various neurotrophic factors, age-related changes in trophic factor expression are poorly understood. The present study focused on age-related and gender-specific trophic factor changes in the choroid plexus of lateral ventricles. We also investigated the cellular-level translational changes in the choroid plexus vasculature and epithelium. Further, we examined age-related transcriptional changes in the epithelial tight junction.

## Methods

### Animals

We used C57Bl/6J mice at the ages of 5–6 (Matured Adult), 11–12 (Middle-aged), and 18–24 (Aged) months, with five males and five females in each group, except in the 11–12 months group where only four females were available. Mice were bred in our laboratory (breeders from Jackson Laboratories, Bar Harbor, ME). Mice were maintained on a standard 12-hour light-dark cycle with free access to food and water. All procedures were carried out in strict accordance with the National Institutes of Health guidelines for the care and use of laboratory animals and approved by the USD Institutional Animal Care and Use Committee. Every effort was made to minimize the number of animals used.

### RNA Extraction and Quantitative PCR Analyses

The brains of the experimental mice were carefully dissected, and the hemispheres were separated. Ventricles were gently rinsed with RNAlater stabilization solution, and the CP was carefully dissected under a microscope. According to the manufacturer’s instructions, total RNA from CP was extracted using an RNAqueous micro kit (Invitrogen). The concentration and purity of RNA at 260/280 nm were determined using a NanoDrop ND-1000 spectrophotometer (Thermo Fisher Scientific, USA). 200ng RNA was reverse transcribed into cDNA (Applied Biosystems High-Capacity cDNA Reverse Transcription Kit, USA) using thermal cyclers (Techne Prime). Gene expression analyses were performed by quantitative real-time PCR (applied biosystems QuantStudio 5 using 500 nM of each forward and reverse primers and SYBR green Universal PCR master mix (Gendepot, USA and Applied Biosystems, USA). Primers (Supplementary Table 1) were designed to amplify gene targets using the Primer3 program (https://bioinfo.ut.ee/primer3-0A0/). The expression levels of each gene target were normalized with the housekeeping gene panel, and the fold change of transcription was quantified using the relative quantification 2-ΔΔCt method.

### QIAcuity dPCR

cDNA preparation was performed as previously described. 200ng RNA was reverse transcribed into cDNA in a 20ul reaction volume. After the reaction, cDNA was diluted in 80ul of dd water, and 3ul of diluted cDNA was used for QIAcuity dPCR reactions. QIAcuity dPCR reactions were conducted in the adult and old CP using cDNA generated from common RT reactions. QIAcuity Four 5-plex digital PCR System (Qiagen, Hilden, Germany). Digital PCR reactions consisted of a 12 μL reaction mixture per well containing 4 μL QIAcuity 4x Eva Green Probe PCR master mix (Qiagen), 400 nM of primers (Supplementary Table 1), PCR grade water (Thermo Fisher Scientific, Waltham, MA, USA), and cDNA template from the experimental group. Assembled reactions were transferred into QIAcuity 8k 96-well Nanoplates (Qiagen) for partitioning using the Qiagen Standard Priming Profile, and nucleic acids were amplified under the following conditions: enzyme activation for 2 min at 95 °C and 45 cycles of 15 s at 95 °C and 30 s at 60 °C followed by 60s at 35oC. Partitions were imaged with 200 ms (Green)exposure time, with gain set to 6 for both target channels. The QIAcuity Software Suite (Qiagen, version 2.0.20) was used to determine the copy number.

### Protein isolation from Choroid plexus

The brains of the experimental mice were carefully dissected. Two hemispheres were separated using a surgical blade, and the ventricles were carefully rinsed with ice-cold PBS. Removed the floating CP and transferred it to a 1.5 ml Eppendorf tube. 80ul RIPA buffer with protease and phosphatase inhibitor was added to the Eppendorf tube, followed by 45 minutes of incubation in the ice. Spin the mixture at 14,000 × g for 20 minutes in a 4°C pre-cooled centrifuge. The supernatant was collected in the new centrifuge, and the resulting protein was concentrated using a 10K MWCO pierce concentrator (Thermo Scientific). The protein estimation was done using the BSA method.

### Protein Simple Jess Western analysis

Western blots were also performed using the Protein Simple Jess Western instrument (San Jose, CA). Cell and tissue lysates were prepared as described above. 4ul of protein samples were mixed with a 5x fluorescent master mix (Protein Simple) to achieve a final concentration of 1x master mix buffer according to the manufacturer’s instructions. Samples were then denatured at 95°C for 5 min. All materials and solutions added onto the assay plate were purchased from Protein Simple except primary antibody.10ul of antibody diluent, protein normalizing reagent, primary antibodies, secondary antibodies, chemiluminescent substrates, 3ul of sample, and 500ul of wash buffer were prepared and dispensed into the assay plate. The assay plate was loaded into the instrument, and protein was separated within individual capillaries. Protein detection and digital images were collected and analyzed with Compass software (Protein Simple), and data were reported as an area under the peak, representing the signal’s intensity. For the primary antibody, rabbit polyclonal anti-klotho ((Proteintech, Cat No. 28100–1-AP) and rabbit polyclonal anti-beta-actin (4970S, Cell Signaling Technology, Davers, MA) were used at 1:100 dilution; for the secondary antibody, anti-mouse NIR and anti-rabbit HRP secondary antibodies from Protein Simple were used.

### Immunohistochemistry

Immunohistochemical analysis was carried out in fresh, frozen mouse brains. Mouse brains were dissected carefully and quickly frozen using dry ice. Immunohistochemical studies were performed in 17 um cryocut coronal brain sections. The brain sections were fixed using precooled histochoice fixative (Sigma) for 10 minutes, followed by blocking with Bovine serum albumin (BSA) for one hour at room temperature. Sections were incubated with different primary antibody (Supplementary Table-2) concentrations in antibody solution (2.5% BSA in PBS) at 4°C overnight. (All the antibody details and concentrations are given in the supplementary table 2) Following primary antibody incubation, slides were washed in 1xPBS three times for five minutes each at room temperature. Slides were then incubated with appropriate fluorescent secondary antibodies (1:500, Alexa-488, and 640, Invitrogen, Carlsbad, CA, USA) in antibody solution for 1.5 h at room temperature. The slides were then rinsed in 1x PBS three times for five minutes each. Finally, Vascular marker Isolectin B4 (1:1000) was added to the slide and incubated for one hour. Slides were washed in 1 xPBS three times, and the cover slipped using VectaMount with DAPI (Vector Laboratories). Sections were viewed, and images were captured using a Nikon Eclipse Ni microscope equipped with DS-Qi1 monochrome, a cooled digital camera, and NIS-AR 4.20 Elements imaging software. The mean pixel value was calculated using Image J version 1.54g

### Blood-CSF barrier permeability analysis

Male mice were anesthetized, and fluorescein isothiocyanate dextran (FITC-dextran-40000da, 100 μl, 50 mg/ml, in saline, Sigma-Aldrich, St. Louis, MO) was injected intravenously in the inferior vena cava vein. After 1 minute, the brains were removed and frozen quickly on dry ice. Coronal sections (20 μm thickness) were taken using the cryostat (Leica Microsystems, CM1860, Buffalo Grove, USA). Fluorescent images of the sections were captured using a Nikon Eclipse Ni microscope and NIS Elements software.

### Data analysis

In all experiments, error bars indicate the standard error of the mean (SEM). The following statistical tests were applied to determine statistical significance: an unpaired two-tailed T-test (GraphPad Prism 8.4.3) was used for QIAcuity dPCR, IHC mean pixel intensity (Image J version 1.54g) and western blot, and One-way ANOVA (GraphPad Prism 8.4.3) was used for the analysis of real-time PCR.

## Results

### Neurotrophic factor expression is significantly altered in the aged choroid plexus.

**Figure 1:**
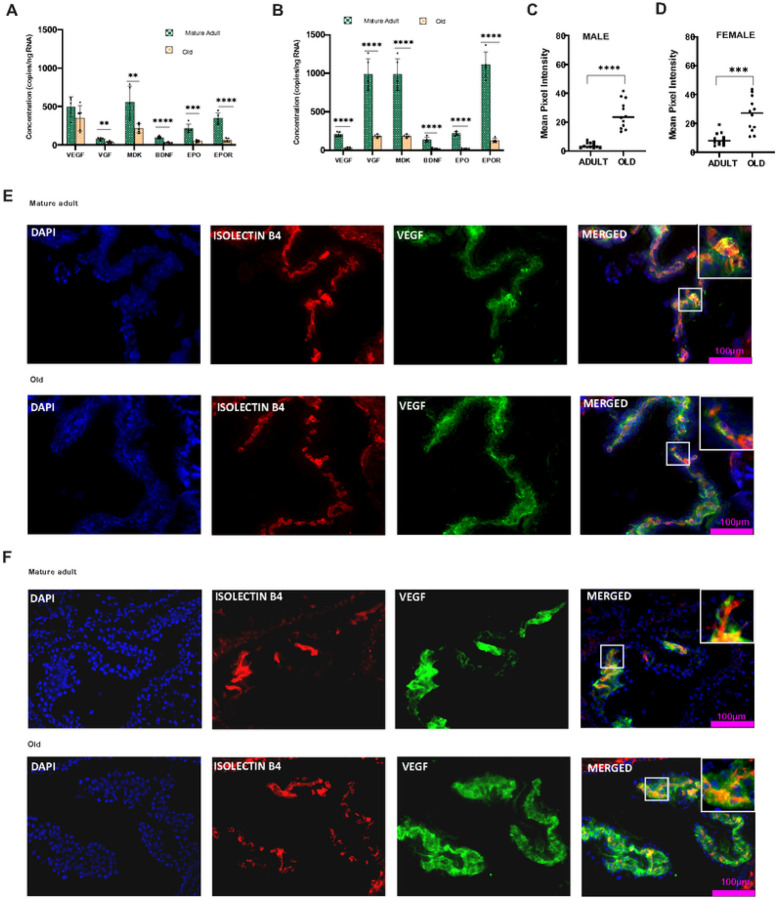
Differential regulation of trophic factors in the aged choroid plexus: **A** Age-related transcriptional level changes in the neurotrophic factors (NTF) in the choroid plexus (CP) of males **B** Age-related transcriptional level changes in the NTF’s in CP of females. QIAcuity dPCR analysis showed that the copy number of neurotrophic factors VGF, MDK, BDNF, EPO, and EPOR was significantly reduced in the old CP of both Males (Figure-1a) and Females. VEGF sowed a gender-specific expression; the concentration was unchanged in males, whereas in females, it showed an age-dependent decline in the copy number. **C** The mean pixel intensity of immunohistochemical analysis showed an increased VEGF staining in the aged CP of males and **D** females. VEGF localization is particularly increased outside CP vasculature of both genders. **E** VEGF (Green)-Isolectin B4 (Red) co-labeling indicates VEGF protein expression in and out of the CP vasculature of males and **F** females. Scalebar-100μm **P<0.01, *** P<0.001, **** P<0.0001

Neurotrophic factors are highly expressed and secreted in the CP and other brain regions. CP secretes several trophic factors into CSF and circulates it to different brain regions [2]. Here, we studied the gender-specific expression patterns of major neurotrophic factors in the murine CP To address the age-dependent transcriptional level changes in murine CP we first analyzed neurotrophic factor gene expression patterns at three different time points: 5–6 m.o. (from now on referred to as mature adult), 11–12 m.o. (middle-aged) and 18–24 m.o. (aged). Further, we confirmed transcript levels using digital droplet PCR. Most of the trophic factors examined showed transcriptional dysregulation in the old CP; some were gender specific. First, we analyzed the transcription pattern of the VEGF gene, which is actively involved in the maintenance of the CP vasculature and has a role in the permeability of blood vessels. VEGF showed a differential transcriptional pattern in males and females. In male CP the relative gene expression showed a consistent VEGF gene expression throughout the lifespan (Supplementary Fig. 1a), and the ddPCR gene expression analysis further confirmed that in males, VEGF mRNA transcription is not affected by aging (Fig. 1A). However, in females, VEGF mRNA expression is significantly (P < 0.001) reduced with age (Fig. 1B), and the reduction is evident from middle age (P < 0.01) onwards (Supplementary Fig. 1b). We used immunohistochemical analysis to determine whether these gene expression changes are mediated at the protein level. Interestingly, unlike gene expression changes, VEGF protein is more highly expressed(P < 0.0001) in the aged CP than in adults in both males (Fig. 1C) and females (Fig. 1D) We also found that VEGF localization was not limited to the choroid vasculature. The expression was also observed in the basal membrane of epithelial cells or stroma (Fig. 1E&1F).

Next, we investigated the expression pattern of Midkine (MDK), another trophic factor highly expressed in the CP MDK is known to provide neurotrophic support and neurite outgrowth [39]. MDK mRNA transcription showed a significant reduction in the aged male (P < 0.001) and female (P < 0.0001) mice (Supplementary Fig. 1A and 1B). The decline in MDK gene expression starts in middle age and continues throughout the later stages of life. ddPCR gene expression (Figure-1A & 1B) also confirmed the reduction of MDK transcripts in CP of older males (P < 0.01) and female mice (P < 0.0001). Then, we turned our attention to BDNF, which is widely expressed in the CNS and is involved in neuronal survival, development, and synaptic plasticity [40, 41, 42]. QIAcuity dPCR analysis showed a lower copy number of BDNF (Fig. 1A) in CP than other trophic factors, indicating reduced transcription of BDNF mRNA in the CP However, an age-dependent reduction is evident in BDNF mRNA levels (Supplementary Figs. 1A and B) in the CP of both males (P < 0.0001) and females(P < 0.001). Another important trophic factor, VGF, showed an age-dependent reduction in VGF gene transcription (Fig. 1A&B) in males (P < 0.001) and females (P < 0.0001). Interestingly, females exhibit a higher copy number than males. Erythropoietin (EPO), a neurotrophic and neuroprotective cytokine, exhibits a significantly lower copy number (Figure-1A &B) in the CP of aged males (P < 0.001) and female mice (P < 0.0001). EPO acts through its classical receptor, the erythropoietin receptor (EPOR). EPOR copy number is significantly decreased (Figure-1A &B) in the aged CP of both males (P < 0.0001) and females (P < 0.0001), indicating reduced EPO signaling in the later stages of life.

### IGF expression decreased in the choroid plexus of aged mice.

**Figure 2:**
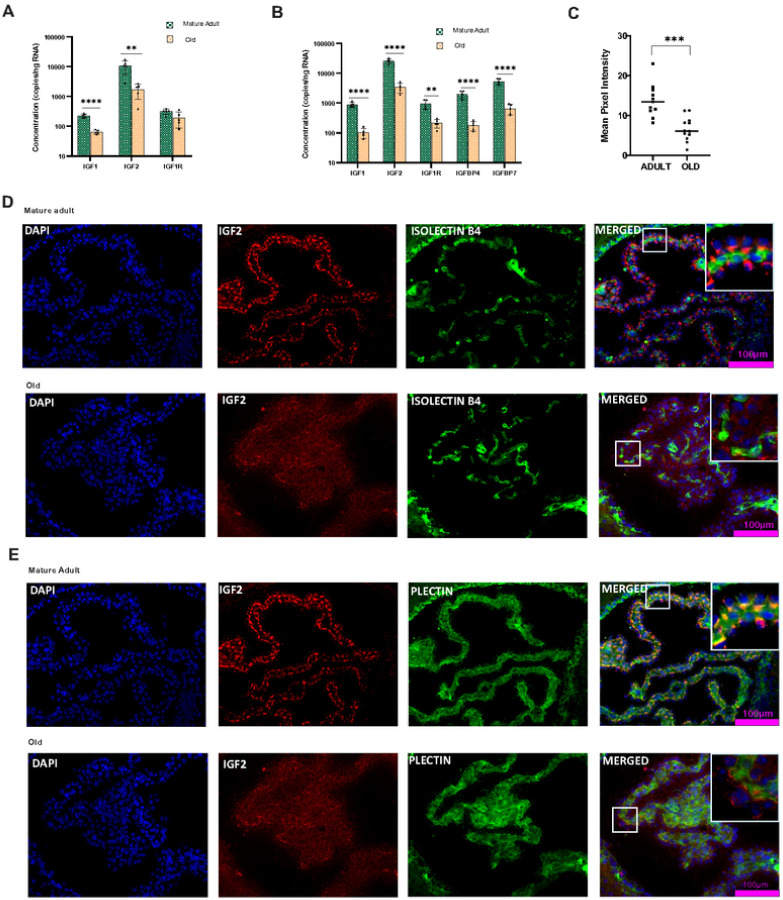
Insulin-like growth factor expression is reduced in aged choroid plexus. **A**, IGF transcription was significantly altered in the aged CP of males and **B** females. IGF1 and IGF2 mRNA Copy numbers showed a significant reduction in both genders, indicating reduced gene expression. IGF1R expression showed a gender-specific expression, IGF1R expression was unchanged in the males and reduced in the females. IGFBP4 and IGFBP7 in the females showed a reduction in copy number, indicating a reduction in the gene expression. **D** IGF2 (Red)-IsolectinB4 (Green) co-labeling showed that IGF2 is absent in choroidal vasculature (Figure 2d). **E**The co-labeling study using plectin (Green) showed that IGF2 (Red) is expressed in the basolateral side of choroidal epithelial cells of male mice. **C** The reduction of IGF2 Protein expression is evident from the mean pixel intensity of IHC images. Scalebar-100μm**P<0.01, *** P<0.001, **** P<0.0001

Insulin-like growth factors (IGFs) are essential growth-promoting peptides that act as endocrine, paracrine, and autocrine factors. IGF signaling plays a crucial role in controlling aging and life span in invertebrates [43, 44]. To analyze IGF signaling in aged mice, we first examined the mRNA of the IGF1 gene. The relative expression of IGF1 mRNA showed differential expression patterns in the aged males and females. We found a significant down-regulation (P < 0.0001) of IGF1 mRNA expression in the CP of aged females. Age-related changes were significant (P < 0.0001) from middle age onwards. ddPCR (Fig. 2b) (P < 0.0001) gene expression confirmed the altered transcription of IGF1 mRNA in aged females. Even though the relative expression (Supplementary Fig. 1C) appeared unchanged in male CP absolute gene expression showed (Fig. 2A) a significant reduction (P < 0.001) in IGF1 transcript levels.

IGF-II is a significant growth factor in the brain and is involved in memory consolidation. IGF-II transcription in the brain is mainly localized at the CP and leptomeninges [45]. Quantitative PCR analysis showed an age-dependent reduction in IGF-II gene transcription in the CP of both males(P < 0.001) and females (P < 0.0001) (Supplementary Fig. 1C & 1D). The reduction was evident from middle age onwards. QIAcuity ddPCR further confirmed the reduced concentration of IGF-II transcripts in aged CP of males (P < 0.01) and females (P < 0.0001) (Fig. 2A&2B). Further, we focused on age-related protein-level changes in IGF- II expression in the CP epithelium and vasculature. The co-labeling study showed that in adult CP, IGF-II protein is expressed in the epithelial cells, particularly in the basal membrane of epithelial cells (Fig. 2E & Supplementary Fig. 3B). The vasculature of the CP is free from IGF-II expression (Fig. 2D & Supplementary Fig. 3A). Natural aging dramatically reduced IGF-II protein expression (Fig. 2C& Supplementary Figure C) in the choroid epithelium of both males(P < 0.0001) and females (P < 0.002).

IGF1 and IGF2 act through the IGF receptor, and the IGF1R gene expression showed a differential expression in both males and females (Fig. 2A&B). Aging did not influence IGF1R gene expression in the male CP (Fig. 2A & Supplementary Fig. 1C), whereas, in females, both the absolute (P < 0.01) and relative (P < 0.001) expression indicate a significant reduction in IGF1R gene transcription (Fig. 2B & Supplementary Fig. 1D). The IGF peptides have a short lifespan unless they are bound by specific binding proteins that transport them in circulation and deliver them to specific tissues. IGFBPs are found throughout the body in various fluids and tissues [46, 47]. IGFBP has binding affinities for IGF-I and IGF-II comparable to the ligands for IGF-IR. Most IGFBPs inhibit IGF-induced cell growth by binding to IGFs and acting as a time-release mechanism. Here, we analyzed the transcription pattern of IGFBP4 and IGFBP7. Quantitative PCR gene expression analysis showed that IGFBP4 and IGFBP7 expression in CP is gender-specific; in males, the gene expression remains unchanged (Supplementary Fig. 1c), and in females (Supplementary Fig. 1D), both IGFBP4 (P < 0.001) and IGFBP7 (P < 0.0001) expression significantly reduces as age progresses. Gene expression study using QIAcuity dPCR (Fig. 2B) confirms the age-induced reduction (P < 0.0001) in the IGFBP transcription.

### Longevity factor Klotho is reduced in the aged choroid plexus.

**Figure 3:**
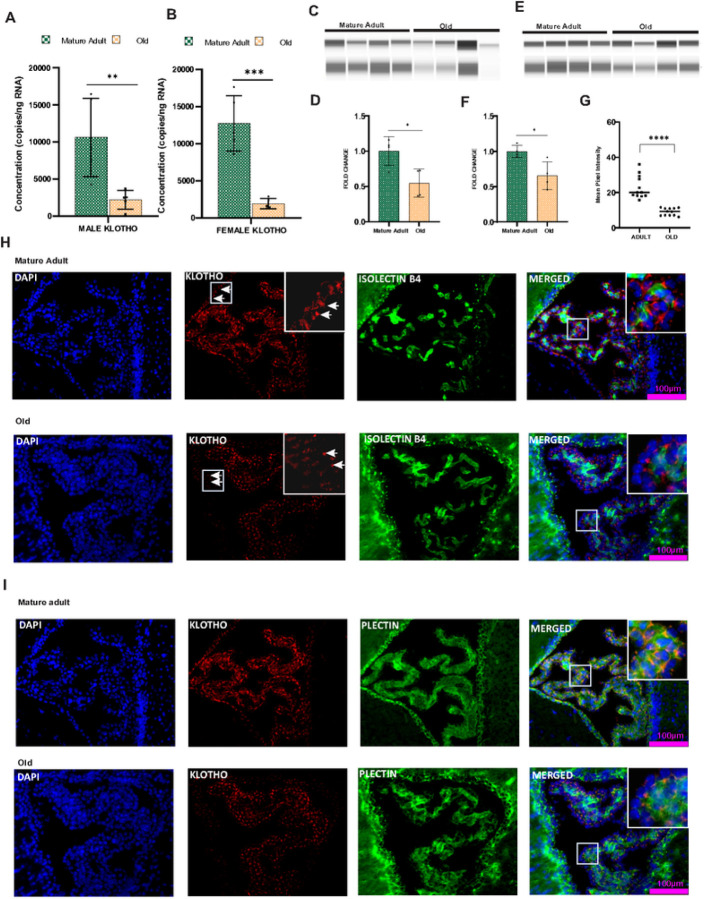
Longevity factor klotho expression was significantly altered in the aged choroid plexus of both genders: Age-related transcriptional level reduction was observed in the CP of **A** Males and **B** Females. A quantitative analysis of Klotho protein was conducted using Jess capillary separation in the CP of **C** males and **E** females. Expression was normalized using β actin. Klotho protein expression was significantly reduced in aged CP of **D** males and **F** females. **H**Klotho (Red) -IsolectinB4 (Green) co-labeling confirmed the absence of Klotho from Choroid vasculature. **I** Pectin (Green)was used to mark the epithelial cells, and the basolateral sides of the choroid epithelial cells are rich in Klotho (Red) protein. **G** The mean pixel intensity of IHC images confirmed the protein level reduction of klotho in aged male CP Aging also reduced klotho staining in the ependymal cells in the ventricles (marked with white arrows). Scalebar-100μm. * P<0.05**P<0.01, *** P<0.001, **** P<0.0001

α-Klotho is a glycosylated transmembrane protein that has been extensively studied as an anti-aging protein. Klotho is expressed highly in the choroid plexus, and its precise functions are largely unknown. We examined the gender and age-specific expression pattern of klotho in the CP Klotho showed a reduced copy number (Fig. 3A&B) in the aged CPs of both males (P < 0.01) and females (P < 0.001), indicating an age-related reduction in Klotho gene expression. The reduction (Supplementary Fig. 1B) was evident in females (P < 0.001) from middle age onwards.

We extended our investigation to examine whether the changes in the mRNA level are reflected at the protein level. First, we used Western blot analysis to examine membrane-bound Klotho protein expression in the aged CP of both genders (Fig. 3C& 3E). Klotho protein expression revealed an age-related reduction (Fig. 3D& 3F) in the CP of males (P < 0.05) and females (P < 0.05). Next, we used IHC analysis to identify age-related changes in klotho protein expression patterns in the choroid epithelium and vasculature. Co-localization of Klotho with plectin indicates that Klotho protein expression is mainly concentrated in the choroid epithelial cells (Fig. 3I & Supplementary Fig. 4B). The apical part of epithelium lacks klotho expression, whereas the basolateral side is rich in klotho protein. Klotho is not co-labeled with isolectin B4, indicating the absence of Klotho protein in the choroid vasculature (Fig. 3H & Supplementary Fig. 4A). IHC analyses clearly indicate an age-dependent reduction (P < 0.0001) in klotho protein expression in CP of males (P < 0.0001) and females (P < 0.001) (Fig. 3G & Supplementary Fig. 4C). Reduced klotho protein expression was also observed in the ventricular lining of the old mice.

### Water channel protein AQP1 expression is altered in the aged choroid plexus.

**Figure 4:**
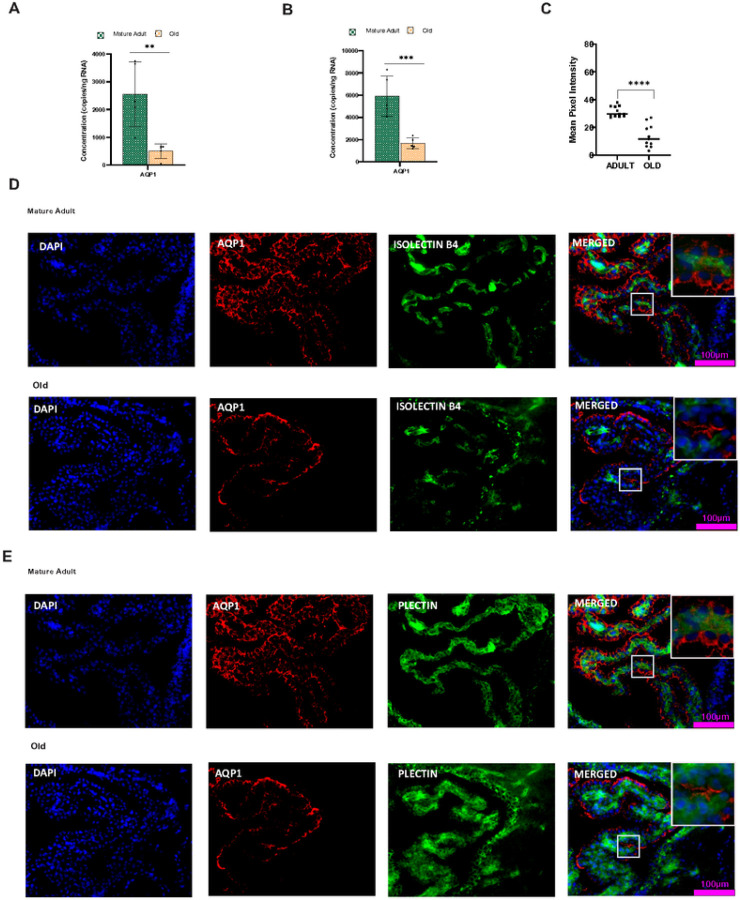
Water channel protein AQP1 expression is reduced in the aged choroid plexus. Aging significantly reduces the water channel protein AQP1 gene expression in the CP of males and **B** females. **D** Aqp1-Isolectin B4 co-labeling confirmed the absence of Aqp-1 protein expression in the adult and old CP vasculature. **E**AQP1-Plectin co-labeling study indicates Aqp1 localization in the apical part of the choroid epithelial cells. **C** The mean pixel intensity of IHC images showed a reduction in AQP1 Protein expression in the aged CP of males and females. Scalebar-100μm **P<0.01, *** P<0.001, **** P<0.0001

A primary function of choroid epithelium is the production of CSF. Aquaporins (AQPs) are a family of small transmembrane proteins that facilitate water transport across plasma membranes during CSF production. AQP1 is the primary water channel expressed in the choroid plexus epithelium. We examined the age-dependent transcriptional changes in the male and female CP AQP1 gene expression (Supplementary Fig. 2A& 2B) is significantly lowered in the aged CP of both males (P < 0.001) and females (P < 0.0001). The reduction was evident from middle age onwards. The ddPCR analysis further confirmed the low copy number in the aged CP (Fig. 4A&B), indicating a significantly reduced concentration of AQP1 transcripts in males (P < 0.01) and females (P < 0.001). The protein expression analysis using IHC indicates that, like AQP1 mRNA transcription, AQP1 Protein expression also showed an age-dependent reduction (P < 0.0001) in the CP epithelium of aged males and females (Fig. 4E & Supplementary Fig. 5C). IHC analysis indicates that the AQP1 protein is absent in the vasculature (Fig. 4D & Supplementary Fig. 5A) and present only in the ventricular-facing surface of the choroid plexus epithelium (Fig. 4E & Supplementary Fig. 5B).

### Aging-altered tight junction protein expression compromises tight junction integrity.

**Figure 5:**
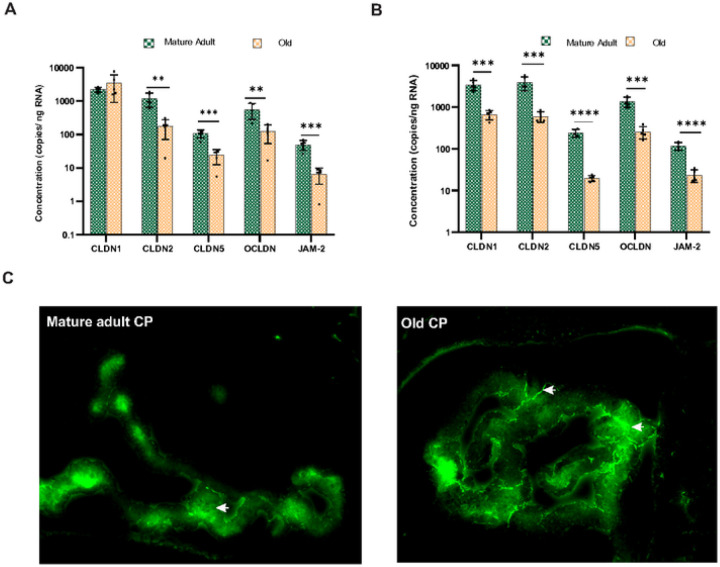
Tight junction proteins are significantly altered in the aged choroid plexus. **A**Tight junctional protein gene expression showed a differential expression in the aged male CP CLDN-1 mRNA transcription was unchanged along with reduced expression of CLDN-2, CLDN-5, OCLDN, and JAM2. **B** In females, CLDN-1, CLDN-2, CLDN-5, OCLDN, and JAM2 transcription was significantly reduced. **C** BCSF barrier permeability was assessed by injecting dextran average mol wt 40000 intracardially. In adult CR fluorescence is mainly observed in the choroid vasculature and basolateral side of the epithelium, indicating that the tight junction prevents the diffusion of FITC dextran to the ventricles (Figure 5c). In aged mice, fluorescence can be observed in and out of the apical side of endothelial cells, indicating that dextran 40000 was able to cross the tight junction. **P<0.01, *** P<0.001, **** P<0.0001

The distribution of tight junctions in the choroid plexus differs from other brain regions. Choroid vasculature is fenestrated and lacks tight junctions [48]. The epithelial cells are connected by tight junctions in the apical region and maintain BCSF integrity [49]. Claudins are one of the most crucial tight junction proteins in the choroid epithelium. In males, real-time PCR analysis showed significant downregulation (P < 0.05) of CLDN1 gene expression in the middle-aged CP and the gene expression came back to normal in the aged CP (Supplementary Fig. 2C). In female CP CLDN1 m RNA transcription showed a trend toward decrease but was not statistically significant (Supplementary Fig. 2D). QIAcuity ddPCR analysis showed a consistent copy number of CLDN1 transcripts in the adult and aged male CP (Fig. 5A). However, the copy number in females was significantly (P < 0.001) reduced in aged CP (Fig. 5B). CLDN2 and CLDN5 mRNA expression were significantly (P < 0.0001) reduced in the aged CP of females (Supplementary Fig. 2D). ddPCR analysis further confirmed the reduction in CLDN2 and CLDN5 gene copy numbers (Fig. 5B). In males, CLDN2 (P < 0.0001) and CLDN5(P < 0.001) gene expression were significantly downregulated in the aged CP (Supplementary Fig. 2C). QIAcuity ddPCR further confirmed the same pattern of reduction in CLDN2 and CLDN5 gene expression (Fig. 5A). The mRNA expression of JAM2 is significantly downregulated in the aged CP of both males (P < 0.001) and females (P < 0.0001) (Fig. 5A & 5B, Supplementary Fig. 2C &2D). DdPCR confirms the reduction in JAM2 mRNA gene expression. To study whether the altered tight junction transcription affects the BCSF barrier integrity, we injected FITC-Dextran (MW 40000) intravenously. Fluorescent imaging showed that barrier permeability was intact in adult CP and fluorescence was restricted within the basolateral side (Fig. 5C). However, dextran leakage can be observed from the apical membrane in the aged CP indicating that BCSF integrity is compromised.

#### Aging altered Transport protein Transthyretin gene expression

**Figure 6:**
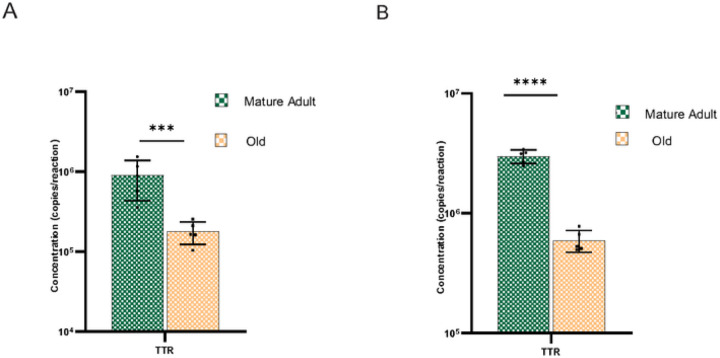
Transthyretin gene expression was significantly downregulated in the aged CP of males and females. **A** Thyroid hormone distributor protein TTR gene expression in the aged CP of males and **B** females. TTR transcription was significantly downregulated in the aged CP of both genders. *** P<0.001.

Transthyretin (TTR) is the major protein synthesized by the choroid epithelial cells and is expressed at remarkably high levels. Functionally, TTR binds and distributes thyroid hormones (THs) in the blood and cerebrospinal fluid [50]. Here, we examined the age and gender-specific changes in the TTR gene transcription. Relative gene expression showed that aging significantly accelerated the downregulation of TTR mRNA (Supplementary Fig. 6E &6F) in the CP of males (P < 0.001) and females(P < 0.001). The ddPCR further confirmed the reduced TTR expression (Fig. 6A &6B) in aged mice.

## Discussion

The CP is known for its role in the production of CSF but still remains a relatively understudied structure in the CNS. CSF contains proteins and growth factors that play roles in CNS development, and these growth factors may be brain-derived or produced by the CP In the present study, we analyzed the age and sex-dependent transcriptional changes in trophic factors, tight junctional proteins, water channel protein AQP1, and the antiaging protein klotho. CSF is mainly composed of 99% water, and the remaining 1% is accounted for by proteins, ions, neurotransmitters, and glucose [51]. A high-water permeability of the BCSF barrier is essential for the optimal production of the CSF [52, 53], and this is met by the abundant expression of water channels AQP4 and AQP1. CSF enters from the perivascular spaces surrounding arteries into the brain parenchyma through the AQP4 water channels in the astrocytic end-feet [54]. AQP1 is found in the apical membrane and is essential for water transport across the CP We observed an age-related reduction in the transcription of AQP1 mRNA in the CP of both males and females. Transcriptional level alterations in AQP1 were evident from middle-aged CP and continued till later stages of life. Both males and females showed a similar pattern of gene expression. The reduction in AQP1 transcription was reflected at the protein level, showing reduced AQP1 protein expression in the aged CP AQP1 localization was only observed in the apical region of cuboid epithelial cells, which is in accordance with the previous studies [55, 56, 57, 58]. More interestingly, we found that the AQP1 water channel is absent in the CP vasculature. AQP1 allows water to follow the osmotic gradient and is also involved in maintaining the osmotic permeability of the apical membrane. AQP1-null mice possess 56% lower CSF pressure and a 20% reduction in CSF production [52]. This age-related reduction in water channel protein AQP1 could be responsible for the reduced production of CSF in natural aging.

Further, we studied the age-related transcriptional changes in the TJ proteins of choroid epithelium. The regulation of BCSF barrier permeability and integrity is a key role of choroid epithelial tight junctions. These apical tight junctions regulate the paracellular diffusion of water-soluble molecules through this barrier. Unlike the BBB, CP vasculature is fenestrated and lacks tight junctions to connect the endothelial cells [59, 60, 61, 62]. Hence, the expression of the tight junctional proteins in CP is primarily from the apical tight junctions of the choroid epithelium. Like BBB, the blood–CSF barrier is also highly restricted to soluble tracers and has higher electrical resistance [63]. The tight junction proteins, claudins occludin and zonula occludens are integral to maintaining epithelial integrity, as they greatly limit paracellular permeability and preserve electrical resistance of the epithelial layer in the choroid plexus. Zonula occludens are sub-membrane proteins attaching occludin and claudins to actin filaments [64, 65], while occludin and claudins are transmembrane proteins facilitating contact between epithelial cells [11]. We found that aging impacts the transcription of the epithelial tight junction proteins, and most of the tight junctional proteins showed a reduction in transcription, resulting in increased permeability in both males and females. Interestingly, this tight junctional protein expression reduction was evident from middle age onwards. The reduced transcription of the Tight junction protein in the aged CP compromises epithelial tight junction integrity and can alter the electrical resistance of the BCSF membrane.

The age-dependent increase in choroid epithelial permeability can significantly influence protein concentration in the CSF. Studies have confirmed that aging can cause a rise in the concentration of numerous CSF proteins [66, 38]. CP secretes trophic factors directly to the CSF, which circulates to different parts of the brain [67]. Here, we analyzed the impact of aging on neurotrophic factor transcription in CP of both males and females. VEGF showed a gender-specific gene transcription in the CP VEGF gene expression was unchanged in the aged male CP while in female CP gene expression was significantly reduced. The reduction in the female cp was evident from middle age onwards. Interestingly, unlike the pattern of gene transcription, VEGF protein levels increased in the aged CP of males and females. Typically, VEGF is located in the vasculature, but in the CP expression is observed in endothelial and epithelial cells. Aging increases the translocation of VEGF protein from the vasculature to the surrounding tissues, possibly stroma or the basal membrane of epithelial cells. VEGF is involved in the maintenance of fenestrated vasculature of the choroid plexus and has known roles in vascular permeability [23]. It would be worthwhile to investigate the role of VEGF in epithelial permeability. BDNF, an important trophic factor involved in the neuroplastic changes related to learning and memory, exhibited reduced transcription in the aged CP The reduction is evident from middle age onwards in both males and females. The hippocampal BDNF protein expression does not decline with age, while the age-related reduction is evident in the cerebral cortex and CSF [68, 69]. Reduced CSF BDNF was suggested as a potential mechanism in the cognitive decline observed in older individuals [69]. This reduction in CSF BDNF could be due to reduced transcription of BDNF mRNA in older CP.

In addition to the above growth factors, both IGF-I and IGF2 are produced in the choroid plexus. The production of IGF1 reaches its peak in the postnatal brain to support oligodendrocyte differentiation and myelination, and it decreases thereafter [70]. An age-related reduction in hepatic IGF1 production leads to a decline in circulating IGF1, resulting in impaired neurovascular coupling responses in older adults [71]. IGF1 and IGF2 can cross the BBB, so the reduction in the circulating levels can influence the concentration of IGFs in the CSF and CNS. Here, we analyzed the transcription of IGF mRNA in the aged CP of both males and females. Females showed an age-dependent reduction in IGF1 transcription from middle age onwards, but in males, expression was unchanged in the middle-aged cp and significantly reduced thereafter. Even though IGF2 is abundantly expressed in the CNS, only CP leptomeninges, and parenchymal microvasculature produce IGF2 [72]. In a healthy brain, CP secretes IGF2 directly to CSF, and CSF distributes this IGF2 to different brain regions. IGF2 is critical for preserving neural stem cells (NSCs) in the adult hippocampus [73] and also plays a vital role in adult neurogenesis, memory formation, neuronal growth, and neuroprotection [74]. A previous study conducted in sheep by Chen [75] and co-workers reported that IGF2 mRNA expression in CP did not change with age. Our results in mice are in variance with these findings. We observed a significant reduction in IGF2 mRNA transcription in the CP of aged males and females. The protein level analysis using IHC also confirmed the age-related reduction in the choroid epithelial cells. CP vasculature lacks IGF2 localization, indicating IGF2 bioactivity is observed only in epithelial cells. Numerous reports suggest reduced IGF2 protein in aged mice can lead to memory deficit and cognitive impairment [76, 77, 78]. One possibility is that reduced protein expression in the hippocampus could be due to age-related transcriptional changes in the CP IGF1 and IGF2 act through their corresponding receptors, IGF1 R and IGF2R [79]. The reduced IGF1R transcription in aged CP may be due to the age-related reduction in IGF1 protein. IGF signaling is regulated by a family of specific IGF-binding proteins (IGFBPs). We observed a gender-specific expression of IGFBP 4 and IGFBP 7 (IGFBP-rp1) in aged CP; IGFBP4 and IGFBP 7 mRNA transcription remained unchanged in male CP whereas in female CP reduction in the IGFBP4 and IGFBP 7 transcription was observed. This reduction in IGFBP could be due to the reduced availability of IGF proteins in the aged CP.

Klotho is known to regulate several pathways involved in aging, including Wnt signaling, insulin signaling, and intracellular pathways, including p53/p21, cAMP Protein kinase C, and TGF B [80, 81, 82, 83]. In mice, the overexpression of the klotho gene extends life span, whereas mutations to the klotho gene reduce life span [84, 85, 86]. In humans, serum levels of Klotho, produced from kidneys, decrease after 40 years of age [87, 88, 89]. In CNS, the main source of klotho is the CP previous studies from our lab, using in situ hybridization, showed that klotho protein is abundantly localized in the CP [90]. We observed a reduction in the transcription of klotho mRNA in the aged CP of males and females. The reduced transcription is reflected at the translation level as well. WB and IHC analyses point towards reduced transmembrane klotho protein secretion and localization in the epithelial cell’s basal membrane. In CSF, soluble klotho levels decrease with age, and patients with AD showed considerably lower klotho levels compared to healthy elderly individuals. Since the klotho does not cross the BBB [91]. CP is the primary source of klotho for the brain. This reduction in CSF klotho level in the elderly is likely due to the altered klotho mRNA transcription and translation in the CP.

Our results highlight transcriptional level changes in the CP during aging. The age-related transcriptional changes are somewhat similar in the CP of males and females. Altered transcription of the water channel protein AQP1 and TJ proteins could be a contributing factor to the reduced CSF production in natural aging. Importantly, aging decreases the expression of neurotrophic factors, and longevity factor Klotho can play a role in regulating the aging of the brain. In the later stages of life, the CP-CSF axis shows a decline in all aspects of its function, including CSF secretion [92, 33, 93], barrier, and secretory functions. This decline in function could increase the risk of developing late-life diseases and cognitive deficits.

## Abbreviations

AQP1: Aquaporin1
AQP4: Aquaporin4
BBB: Blood-Brain Barrier
BCSFB: Blood Cerebrospinal Fluid Barrier
BDNF: Brain-derived neurotrophic factor
CLDN-1: Claudin1
CLDN-2: Claudin2
CLDN5: Claudin5
CNS: Central Nervus System
CP: Choroid Plexus
CSF: Cerebrospinal Fluid
DDPCR: Digital droplet Polymerase chain reactor
EPO: Erythropoietin
EPOR: Erythropoietin receptor
IGF1: Insulin-like growth factor1
IGF1R: Insulin-like growth factor1 receptor
IGF2: Insulin-like growth factor2
IGFBP: Insulin-like growth factor binding protein
IGFBPrp: Insulin-like growth factor binding protein-related protein
IHC: Immunohistochemistry
JAM: Junctional adhesion molecule
NSCs: Neural stem cells
NTF: Neurotrophic factors
TGF β: Transforming growth factor-β
THs: Thyroid hormones
TJP: Tight junctional Protein
TTR: Transthyretin
VEGF: Vascular endothelial growth factor
VGF: Nerve growth factor inducible
